# Estimating Bat and Bird Mortality Occurring at Wind Energy Turbines from Covariates and Carcass Searches Using Mixture Models

**DOI:** 10.1371/journal.pone.0067997

**Published:** 2013-07-03

**Authors:** Fränzi Korner-Nievergelt, Robert Brinkmann, Ivo Niermann, Oliver Behr

**Affiliations:** 1 oikostat GmbH, Ettiswil, Switzerland; 2 Swiss Ornithological Institute, Sempach, Switzerland; 3 Leibniz Universität Hannover, Institut für Umweltplanung, Hannover, Germany; 4 Freiburg Institute of Applied Animal Ecology, Freiburg, Germany; 5 Friedrich-Alexander-Universität Erlangen-Nürnberg, Institut für Tierphysiologie, Erlangen, Germany; University of Regina, Canada

## Abstract

Environmental impacts of wind energy facilities increasingly cause concern, a central issue being bats and birds killed by rotor blades. Two approaches have been employed to assess collision rates: carcass searches and surveys of animals prone to collisions. Carcass searches can provide an estimate for the actual number of animals being killed but they offer little information on the relation between collision rates and, for example, weather parameters due to the time of death not being precisely known. In contrast, a density index of animals exposed to collision is sufficient to analyse the parameters influencing the collision rate. However, quantification of the collision rate from animal density indices (e.g. acoustic bat activity or bird migration traffic rates) remains difficult. We combine carcass search data with animal density indices in a mixture model to investigate collision rates. In a simulation study we show that the collision rates estimated by our model were at least as precise as conventional estimates based solely on carcass search data. Furthermore, if certain conditions are met, the model can be used to predict the collision rate from density indices alone, without data from carcass searches. This can reduce the time and effort required to estimate collision rates. We applied the model to bat carcass search data obtained at 30 wind turbines in 15 wind facilities in Germany. We used acoustic bat activity and wind speed as predictors for the collision rate. The model estimates correlated well with conventional estimators. Our model can be used to predict the average collision rate. It enables an analysis of the effect of parameters such as rotor diameter or turbine type on the collision rate. The model can also be used in turbine-specific curtailment algorithms that predict the collision rate and reduce this rate with a minimal loss of energy production.

## Introduction

Wind energy production is growing rapidly in many countries. It is widely accepted as a renewable source of energy that does not entail the ecological problems inevitably associated with other sources of energy, particularly fossil fuels and nuclear energy. In Germany, the number of wind energy turbines has increased from 1,200 in 1992 to 23,030 in 2012 [1]. However, there is concern that the growing production of wind energy is accompanied by new conservation issues, in particular the mortality of birds and bats through direct impact with rotor blades (e.g. [2]–[5]). Further concerns regard the loss of nesting or foraging habitat, visual and sound impact, and aesthetic landscape aspects [6]. An increase in mortality can have a severe impact on vulnerable bird and bat populations [7]–[10]. Therefore, mortality should be quantified and studied in relation to landscape and meteorological parameters or technical parameters of wind turbines in order to predict and reduce the collision rate.

Cases of birds colliding with wind turbines have been widely documented and studied since the late 1990s [11], [12]. More recently, alarming numbers of bat fatalities have been documented at wind energy facilities in different parts of the world [12]–[15]. Carcass searches have often been used to quantify the impact of existing wind turbines on bats and birds. However, a number of studies have shown that carcass searches may vastly underestimate the actual number of animals killed when detection biases are not taken into account [16]–[18]. To overcome the problem of detection biases several functions have been developed to estimate the probability *p* that a searcher finds an animal which has collided with a rotor blade [13], [17]–[21]. These functions are based on the fraction of the area below the turbines being searched, the theoretical [22] or empirical [20] spatial distribution of carcasses, persistence rate, and searcher efficiency. The number of carcasses found *C* is often divided by the estimated detection probability to obtain an estimate for the number of fatalities 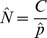 [17], [19]. Alternatively, the theorem of Bayes can be used to obtain a probability distribution of the number of collisions given the detection probability and the number of carcasses found [18]. Henceforth, we will call these methods “corrected counts”. However, the uncertainty of the estimated number of collisions resulting from these formulas is high, particularly when the number of carcasses found is low [18], as is often the case in Central Europe. Therefore, we propose to combine carcass search data with additional predictors for the collision rate in a single model to estimate the number of collisions at wind turbines. To achieve this, we adapted the model presented by A. Royle [23]. The model consists of two sub-models, one for the collision and one for the observation (i.e. carcass search) process. In the collision sub-model, the collision rate is modelled by predictors such as wind speed, rotor diameter and animal density measurements (e.g. acoustic bat activity).

As an example, we applied the model to estimate bat collision rates at 30 wind turbines in Germany. We used acoustic bat activity and wind speed (both measured at the nacelle of turbines) as predictors for the number of bat collisions. Acoustic bat activity has been shown to be a good predictor of the bat collision rate [12], [24], [25]. Wind speed primarily determines the speed of the rotor blade and is therefore expected to correlate strongly with the collision rate. In the observation sub-model, the number of bat collisions was related to the number of bat carcasses found, taking the carcass detection probability *p* into account. We present two versions of the observation sub-model, a one-level and a three-level observation model. The two models represent different solutions of the trade-off between reducing the assumptions at the cost of increasing complexity (see below).

We first assess the bias, precision and predictive power of the mortality estimates from the model using simulated data and compare these with mortality estimates obtained by conventional methods. Subsequently, we apply our model to a real data example collected at 30 wind turbines in Germany [14]. Finally, we discuss potential further applications of the model.

## Materials and Methods

### Data

During the summer of 2007 and 2008 a total of 30 wind turbines (12 turbines in 2007 and 18 different turbines in 2008) were sampled at 15 different facilities (2 turbines per facility) during an average of 68 (range 12–83) nights per turbine. The sample sites covered a variety of geographical regions which stretch from the coastal plains in the north to low mountain ranges in the west and east of Germany. All turbines were from the same manufacturer (ENERCON) and had rotor diameters between 66 m and 72 m (median 70 m).

At each wind turbine, nocturnal acoustic bat activity was measured continuously during the months July to September using an ultrasound detector (“Batcorder”, Ecoobs, Germany). This detector has been developed to enable an automated recording of ultrasound calls produced by bats. The detectors were calibrated to an identical sensitivity level by the manufacturer and were used at a sensitivity setting of −27 dB in 2007 and −36 dB in 2008, and mounted in the nacelle with the microphone facing downwards to sample the bat activity in the lower rotor-swept zone. The bat species recorded use echolocation calls in an ultrasound range of approximately 17 to 60 kHz for orientation and the detection of prey. We used the number of recordings per night as a measure of bat activity. Each recording contained at least one bat echolocation call, in most cases a short sequence of calls. The identity of the species was determined by the automated call classification software BCDiscriminator (Ver 1.13, EcoObs, www.ecoobs.de, [26]). Recordings identified by the software as bat calls were checked manually and removed from the data-set when they only contained noise signals. We did separate analyses for two different data sets (Table 1).

**Table 1:** Characteristics of the two data sets analysed, including the number of turbines investigated, the total number of turbine-nights, the number of bat call recordings, the total number of carcasses found, and the average wind speed with standard deviation.

year	number of turbines (I)	number of nights	number of recordings	number of carcasses found	average carcass detection probability	Average wind speed in m/s (SD)
2007	12	473	2187	22	0.58	5.2 (1.9)
2008	18	1225	16263	35	0.61	5.5 (1.8)

The area within a 50 m radius below each wind turbine was systematically searched for dead bats every morning (see Table 2 for the number of searches per turbine, [20]). Additional experiments were carried out to estimate the carcass detection probability *p* at each turbine. The estimation of carcass detection probability *p* is not the focus of this article. Our aim is to incorporate into the collision model the carcass detection probability *p* after it has been determined (i.e. we assume that accurate estimates for *p* are available, Table 2). Detailed descriptions of the method to estimate carcass detection probability have been published in [20] and [18] and reviewed in [21]. Here is a short summary of the methods used in our example data. We took into account the proportion of killed bats that have fallen into the search area *a*, carcass persistence probability *s* and searcher efficiency *f*.

**Table 2:** Carcass search data for the 30 turbines sampled.

turbine	year	C_i._	T_i_	a_i_	s_i_	s_i_.lwr	s_i_.upr	f_i_	f_i_.lwr	f_i_.upr	p_i_	p_i_.lwr	p_i_.upr
1	2007	1	23	0.92	0.83	0.87	0.99	0.69	0.63	0.76	0.70	0.50	0.85
2	2007	3	45	0.94	0.82	0.64	0.94	0.70	0.63	0.77	0.71	0.48	0.88
3	2007	7	43	1.00	0.89	0.60	0.93	0.74	0.67	0.79	0.85	0.66	0.97
4	2007	1	14	0.52	0.84	0.58	0.93	0.74	0.67	0.80	0.41	0.30	0.49
5	2007	0	51	0.27	0.79	0.60	0.93	0.66	0.63	0.69	0.19	0.13	0.24
6	2007	0	65	0.62	0.59	0.56	0.91	0.65	0.62	0.68	0.30	0.12	0.48
7	2007	1	37	1.00	0.80	0.69	0.96	0.80	0.74	0.84	0.76	0.63	0.88
8	2007	3	37	0.97	0.71	0.60	0.94	0.77	0.70	0.84	0.63	0.34	0.87
9	2007	1	25	0.53	0.82	0.62	0.93	0.74	0.71	0.77	0.41	0.27	0.50
10	2007	0	25	0.66	0.85	0.64	0.94	0.75	0.72	0.79	0.54	0.39	0.63
11	2007	2	54	1.00	0.93	0.55	0.92	0.59	0.51	0.67	0.88	0.71	0.97
12	2007	3	54	1.00	0.75	0.61	0.93	0.63	0.57	0.69	0.65	0.36	0.90
13	2008	0	72	0.91	0.96	0.59	0.93	0.60	0.56	0.64	0.84	0.74	0.90
14	2008	0	83	1.00	0.84	0.50	0.91	0.56	0.51	0.60	0.75	0.52	0.92
15	2008	0	72	0.94	0.82	0.13	0.74	0.68	0.65	0.72	0.71	0.51	0.86
16	2008	1	72	0.83	0.80	0.48	0.90	0.71	0.68	0.74	0.61	0.42	0.76
17	2008	0	27	0.89	0.84	0.53	0.91	0.66	0.61	0.70	0.69	0.51	0.83
18	2008	0	81	1.00	0.85	0.24	0.82	0.56	0.51	0.62	0.76	0.55	0.93
19	2008	5	77	0.95	0.80	0.65	0.89	0.64	0.60	0.67	0.68	0.46	0.86
20	2008	9	78	0.94	0.83	0.36	0.89	0.63	0.60	0.67	0.71	0.52	0.87
21	2008	5	83	0.81	0.82	0.56	0.92	0.64	0.58	0.71	0.60	0.42	0.75
22	2008	3	68	0.82	0.77	0.56	0.92	0.65	0.59	0.71	0.57	0.37	0.74
23	2008	1	12	0.64	0.47	0.54	0.94	0.69	0.65	0.73	0.24	0.09	0.44
24	2008	3	77	0.89	0.75	0.59	0.95	0.65	0.61	0.68	0.59	0.36	0.78
25	2008	0	65	0.46	0.81	0.79	0.98	0.78	0.72	0.84	0.36	0.26	0.43
26	2008	0	83	0.73	0.80	0.42	0.91	0.76	0.71	0.81	0.55	0.40	0.67
27	2008	3	74	0.96	0.72	0.43	0.88	0.63	0.59	0.67	0.60	0.35	0.82
28	2008	1	68	1.00	0.72	0.43	0.88	0.65	0.61	0.68	0.63	0.38	0.85
29	2008	1	80	0.82	0.74	0.45	0.89	0.78	0.73	0.82	0.57	0.35	0.74
30	2008	3	53	0.82	0.73	0.43	0.89	0.74	0.68	0.80	0.55	0.34	0.73

C_i._: total number of carcasses found during the T_i_ searches carried out at turbine i; a**_i_**: probability that a killed bat fell into the area that was searched; s_i_: probability that a carcass remained on the ground for 24 hours; f**_i_**: average searcher efficiency (probability that a carcass lying in the searched area was found during one search); p_i_: probability that a killed bat was found by a searcher during the study period;.lwr and.upr give the lower and upper limit of the 95% confidence intervals.

To obtain the proportion of killed bats falling into the search area *a* we combined the proportion of area searched in each 10 m distance ring from the turbine with the proportion of bat carcasses falling into the different distance rings. The latter was obtained empirically [20].

To estimate carcass persistence probability *s* at each of the 30 turbines, a total of 630 brown mice carcasses and 32 bat carcasses were placed inside the area searched below the turbines. Each individual carcass was monitored in daily searches until its disappearance over a maximum of 14 days after placement. Carcasses that did not disappear until day 14 were treated as censored data in the subsequent time-to-event analyses using an exponential model to obtain average daily carcass persistence probability per turbine, 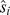 [18], [20].

Searcher efficiency trials were carried out during the entire study period. One person placed either an artificial brown mouse (n = 682), a brown mouse carcass (n = 363) or a bat carcass (n = 37) before the second person started a regular carcass search [20]. The proportion of items found by each person was analysed using generalised linear mixed models with binomial error distribution, the logit-link function, and person as a random factor. Searcher efficiency was estimated separately for three visibility classes (vegetation coverage). Differences in detectability between artificial brown mice, brown mice or bats were negligible. Turbine-specific searcher efficiencies, 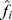, were obtained as weighted averages with weights proportional to the number of searches at a specific turbine per person and the proportion of the three different visibility classes [20].

The proportion of killed bats falling into the area searched (*a_i_*), estimated carcass persistence time (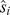), and estimated searcher efficiency (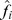) were combined to get turbine-specific estimates of the carcass detection probability 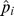 and its standard error using the method given by [18]. At this stage, our data contained turbine-specific carcass detection probabilities averaged over time. However, the model we present below could also account for time-varying detection probabilities.

### The model

#### The model structure

Our model is an adaptation of the N-mixture model developed by A. Royle [23]. Royle’s model was established to estimate animal populations from count data when the probability of detecting an animal is less than one. This model is used to obtain estimates for population sizes that are corrected for observer bias due to the non-detection of parts of the population. Replicated counts of a temporarily closed system (i.e. replicated counts of the same population) allow for an estimation of detection probability and therefore an unbiased estimation of population size [27]–[29]. However, carcasses lying on the ground are not a closed system since they may be removed by scavengers [16], [20]. Therefore, the carcass population available for detection by us is an open population. As a consequence, detection probability cannot be estimated from the number of carcasses detected alone (unless the data meets some specific requirements [30]). We overcome this problem by integrating additional information on carcass detection probability in the sub-model for the observation process using Bayesian methods.

#### Sub-model for the observation process

We present two versions of the sub-model for the observation process: a simple version including one stochastic level and a more complex version including three stochastic levels. In the one-level sub-model for the observation process, the number of carcasses found at turbine *i* on day *t*, *c_it_*, was modelled as a binomially distributed variable with the number of collisions on that day, *N_it_*, as the size parameter and the estimated detection probability, 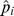, as the success probability (see Table 3 for notations of parameters and variables).
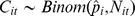



**Table 3:** Notation of parameters, variables and indices.

Name	Description
***Parameters***	
*P*	carcass detection probability: probability that an animal that has been killed by a rotor blade is found by a searcher
*A*	proportion of carcasses lying in the search area: dependent on the spatial distribution of the carcasses and the search area
*S*	daily carcass persistence probability: probability that a carcass remains in the area for 24 hours
*F*	searcher efficiency: probability that a carcass lying in the search area is found by the searcher during one search
***Indices***	
*I*	turbine
*T*	day
*R*	simulation
***Variables***	
*c_it_*	number of carcasses found at turbine *i* at day *t*, “count”
*N_it_*	number of collisions per day, collision rate
*N_i._*	total number of collisions at turbine *i*
*A_it_*	acoustic activity measures, total number of bat calls during night *t* at turbine *i*
*W_it_*	median over night *t* at turbine *i* of the mean wind speed during 10 min intervals
*zA_it_, zW_it_*	standardized (to a mean of zero and a standard deviation of one) acoustic activity and wind speed variables

The model assumes that the animals die during the time interval *t* (of length 24 hours) and that searches take place at the end of the time intervals. Note that the temporal distribution of the collisions within the 24-h time interval depends on the activity pattern of the study species: diurnal birds die more often during the day whereas nocturnal bats die during the night. Subsequently, we will call the time interval “day”, although we realize that when studying nocturnal bats it may be more meaningful to call this interval “night”.

The one-level sub-model assumes a direct relationship between the number of collisions during day *t* and the number of carcasses found after day *t*, i.e. it assumes that all carcasses found during search *t* have been killed during day *t*. Therefore, this model does not account for the possibility that carcasses killed before day *t* can still be found during search *t* if they were overlooked during earlier searches and were not scavenged. The model parameter *N_it_* is estimable, when 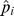 is known or estimated. When 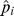 is the point estimate and se(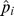) its standard error, the knowledge about *p_i_* can be expressed as a beta-distribution *p_i_*∼*Beta*(*α^p^_i_*,*β^p^_i_*) with mean 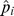 and variance se(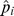)^2^, i.e. with *α^p^_i_* = 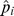
(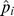
(1-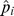)/se(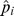)^2^ - 1) and *β^p^_i_* = (1 -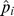)(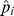
(1 -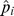)/se(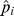)^2^ - 1). These beta-distributions were used as informative prior distributions for the parameter *p_i_* in the model above.

The three-level sub-model for the observation process contains a stochastic part for each of the three steps involved from the collision event to the finding of the carcass: 1) falling into the searched area, 2) remaining on the ground (i.e. not being scavenged), and 3) being found by a searcher. This sub-model allows for carcasses that have not been found during search *t* to be found during later searches. First, the number of fresh carcasses falling into the area searched during day *t* at turbine *i*, *N^fa^_it_*, was modelled as a binomially distributed variable with the number of collisions, *N_it_*, as size parameter and *a_it_* as success probability, where *a_it_* is the proportion of carcasses falling into an area searched.
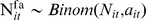



Therefore, the number of carcasses present in the search area before removal by scavengers is the number of carcasses that have remained on the search area from the past, *N^re^_it_*_-1_, minus the number of carcasses found during the last search, *c_it_*_-1_ (since they were removed by the searcher for further investigations), plus the new carcasses killed during day *t*, *N^fa^_it_*

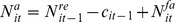
with *N^a^_i_*_1_ = *N^fa^_i_*_1_, assuming that no carcasses were present at the beginning of the study.

Secondly, the number of carcasses remaining until search *t* was modelled as a binomial random variable with the persistence probability *s_it_* as probability parameter:
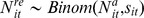



Thirdly, the number of carcasses found during search *t* was modelled as a binomial random variable:
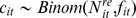
with *N^re^_it_* being the number of carcasses remaining in the search area until search *t* and *f_it_* as the searcher efficiency, i.e. the probability that a carcass which is actually lying in the area searched is found by the searcher during one search.

Estimates for *s* and *f* from Table 2 were used as parameters for the beta-prior distributions for these parameters: *s_it_*∼*Beta*(*α^s^_i_*, *β^s^_i_*) and *f_it_*∼*Beta*(*α^f^_i_*, *β^f^_i_*). The parameters of the beta-distributions were obtained from the means and standard errors of the estimates for *s* and *f* (as described above for the detection probability *p*). In our example, *a_it_* was kept constant per turbine, therefore *a_it_* = *a_i_*, and *a_i_* was assumed to be known without error (Table 2).

#### Sub-model for the collision process

The number of bat collisions during day *t* at turbine *i*, *N_it_* was modelled as a Poisson distributed variable with *λ_it_* as expected value, 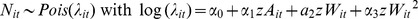
and *zA* and *zW* being the standardised (z-transformed) activity and wind speed measurements respectively.

In our example, the linear predictor contained acoustic activity *A_it_* (i.e. the number of recordings of bat echolocation calls per night, see above), and the median of the mean wind speed during 10 min intervals *W_it_*. Both predictors were measured at the nacelle of the turbine *i* during night *t*. In the turbines studied here, wind speed is related linearly to the speed of the rotor blades until the rotor speed reaches its maximum. Therefore, both linear and quadratic effects were included in the model. We standardised the predictor variables (over the whole data set) to a mean of zero and a standard deviation of one (z-transformation) to increase the speed of the model fitting algorithm. The natural logarithm was used as link function. For the model coefficients *α*_0_, *α*_1_, *α*_2_, and *α*_3_, flat normal distributions with a mean of zero and variance of 100 were used as prior distributions.

#### Parameter estimation

We applied Bayesian methods to estimate the model coefficients. Markov chain Monte Carlo simulations (MCMC) were used to sample from the posterior distributions of the model parameters.

Two Markov chains were run with 20,000 iterations each and the burn-in was set to 10,000 for the one-level observation model. For fitting the three-level observation model, we used 100,000 iterations with a burn-in of 90,000 since convergence was slow in this model. Convergence was assessed by the Brooks-Gelman-Rubin diagnostics [31] and visual inspection of the MCMC history.

The MCMC sampling was done in WinBUGS [32]. The BUGS code of the model is provided in File S1. All programming was done in R 2.15.2 [33] using the package *R2WinBUGS* as an interface to WinBUGS [34].

### Bias and Precision of Mortality Estimation and Predictive Power of the Model

#### Data simulation

To assess the bias and precision of our mortality estimation and to measure the predictive power of the model we carried out a simulation study. Data simulation was based on acoustic bat activity and wind speed measures from our larger 2008 data set including 18 turbines (Table 1). The number of collisions and the number of carcasses found were simulated so that the collision process was imitated as closely as possible. The simulation proceeded as follows: 1) A predefined number of *T_i_* = 100 or 300 pairs of acoustic activity and wind velocity measurements were drawn with replacement from observed values for each of the 18 turbines. This non-parametric bootstrap assured that the correlation between wind speed and acoustic activity measurements, along with the between turbine variance in these measurements were realistic. 2) The values for the model parameters *α^r^*_0_, *α^r^*_1_, *α^r^*_2_, and *α^r^*_3_ were drawn from the joint posterior distribution of these parameters received by the three-level observation model fitted to the data set 2008 (see Table 4), with *r* = 1, …., *R*, the number of simulations. This provided realistic numbers of collisions per day and turbine, which had a mean of 0.046 collisions per day. 3) The expected number of collisions at turbine *i* during day *t* was calculated as *λ^r^_it_* = exp(*α^r^*_0_+ *α^r^*_1_ *zA^r^_it_*+*α^r^*_2_ *zW^r^_it_*+*α^r^*_3_ *zW^r^_it_* ^2^). 4) The virtual “true” number of collisions per day and turbine *N^r^_it_* was drawn from a Poisson distribution with expected value *λ^r^_it_*. 5) The true number of carcasses that had fallen into the search area, *N^fa r^_it_*, was drawn from a binomial distribution *N^fa^^r^_it_*∼*Binom*(*N^ r^_it_*, *s_i_*) with *N^r^_it_* and *a_i_* as parameters. 6) The true number of carcasses that were present in the area searched (before removal by scavengers) was obtained as the sum of the freshly killed animals and the ones that had been killed during earlier days and still remained in the area (i.e. that had not been removed by scavengers or the searcher): *N^a r^_it_* = *N^re r^_it_*_-1_– *c^r^_it_*_-1_+ *N^fa r^_it_*. 7) The number of carcasses remaining in the area until search *t* was drawn from a binomial distribution *N^re r^_it_*∼*Binom*(*N^a r^_it_*, *s_i_*) with *s_i_* being the daily carcass persistence probability at turbine *i*. 8) Finally, the number of carcasses found was simulated from another binomial distribution with *N^re r^_it_* as size parameter and the searcher efficiency *f_i_* as success probability: *c^r^_it_*∼*Binom*(*N^re r^_it_*, *f_i_*). The values *a_i_*, *s_i_* and *f_i_* were taken from our real data set 2008 (Table 2).

**Table 4:** Parameter estimates (mean and 95% credible interval) for our two models (see text) fitted to different data sets (2007, 2008), and the mortality estimates (mean and range of the total number of collisions at all turbines during n nights) based both on the model (second last column), and on the conventional “corrected count” method.

data set and model	n nights	Intercept α_0_	acousticactivity α_1_	wind velocity α_2_	windvelocity^2^ α_3_	mortality estimate from model	correctedcount
2007, 1-level observation model	473	−2.7 (−3.2, −2.1)	0.4 (0.1, 0.7)	−0.2 (−0.9, 0.3)	−0.2 (−0.6, 0.2)	37 (28–49)	38 (27–59)
2007, 3-level observation model	473	−2.3 (−2.9, −1.7)	0.5 (0.1, 0.9)	0.4 (−0.4, 1.3)	−0.7 (−2.0, 0)	38 (29–49)	38 (27–59)
2008, 1-level observation model	1225	−3.5 (−4.1, −3.0)	0.4 (0.1, 0.7)	−1.8 (−3.0, −0.8)	−0.8 (−1.6, −0.3)	56 (46–70)	57 (42–89)
2008,3-level observation model	1225	−4.1 (−5.3, −3.3)	0.5 (0.2, 0.8)	−2.9 (−5.6, −1.2)	−1.3 (−2.6, −0.4)	57 (46–71)	57 (42–89)

This data simulation was repeated *R* = 50 times for each of the 2 different sample sizes *T_i_*. Average true number of collisions per turbine was 4.5 (range: 0–20) with the sample size *T_i_* = 100 and 14.2 (range: 0–48) when sample size was *T_i_* = 300.

The R-code for the data simulation is given in File S2.

#### Bias estimation and predictive power

For each simulated data set, one turbine was randomly selected to serve as a test data set, i.e. it was excluded from the model fitting (termed turbine no. 18). The data of the remaining 17 turbines (training data) was used to fit the one-level and the three-level model. To assess the bias of the fatality estimates from our model, model estimates for the total number of fatalities for each turbine, 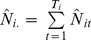, were obtained from both models for the 17 turbines of the training data set. These estimates were compared to the “true” (simulated) number of collisions (see below). To assess the predictive power of the models, the wind and acoustic activity measurements of the test data set (i.e. from the excluded turbine no. 18) were used to predict the number of collisions for turbine no. 18. To do so, we used the predictive distributions of the number of collisions per day 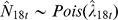 with log() = 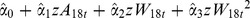. The model parameters 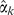 (k = 0, …, 3) were taken from the model fitted to the training data (the 17 turbines). The estimated total number of collisions at turbine no. 18, 
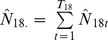
, was then compared with the “true” simulated number of collisions at this turbine.

The bias was calculated as the ratio between the estimated and true number of collisions: 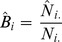


To compare the model estimates with estimates obtained with the conventional method of “correcting” the total number of carcasses found using the carcass detection probabilities, we also calculated these “corrected count” estimates. We applied the Bayes theorem to obtain the estimated mortality as described in [18]. Estimation errors in the detection probability 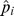 were propagated to the mortality estimate using Monte Carlo simulation [18]. We implemented this procedure in the function estimateN of the package carcass (www.r-project.org/CRAN).

### Application of the Model to Real Data

We fitted both the one-level and the three-level model to the real data sets of the two years sampled (Table 1). For each turbine in the data sets, we estimated the total number of collisions, 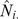. Additionally, we estimated the total number of collisions summed over all turbines during the study period 
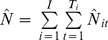
 with *I* = the number of turbines and *T_i_* = the number of nights sampled per turbine. These estimates were compared with the conventional “corrected count” estimate.

The goodness of fit was assessed by predictive model checking. The distribution of the observed number of carcasses found, *c_it_*, was compared with the distribution of numbers of carcasses found that were simulated from the model, i.e. the posterior predictive distribution.

## Results

### Bias and Precision of Mortality Estimation and Predictive Power of the Model

**Figure 1:**
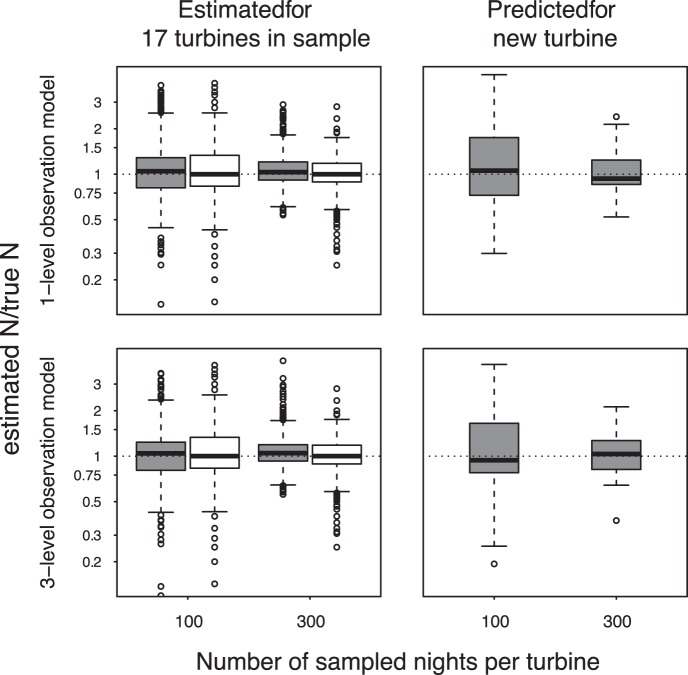
Ratio of the estimated and the true number of collisions per turbine for 50 simulated data sets. 17 turbines were used to fit the model (1700 data points for each box, left panels), collisions were then predicted for a new turbine not used to fit the model (50 data points for each box, right panels). The y-axis is log-scaled so that equivalent proportional increases and decreases result in the same change on the y-axis. Grey boxes indicate model estimates using the one- (upper panels) and three-level (lower panels) observation model (see text). White boxes give the conventional “corrected count” estimates (see text) using only carcass count data and estimates for carcass detection probability. The data has been simulated for two settings with different sample sizes (100 and 300 nights per turbine). The average total numbers of collisions per turbine for these two settings were 4.5 and 14.2 bats respectively. Bold horizontal line = median, box = 50% range of the data, whiskers = last value within 1.5 times the interquartile range, circles = data points further away.

The median ratio between the estimated and the (simulated) true number of collisions per turbine was close to one for both models in all settings (Fig. 1, left panels). Also, the predictions for the new turbine seemed to be unbiased (Fig. 1, right panels). This indicates that the model produces unbiased mortality estimates for the turbines sampled as well as for new turbines of the same type and in a similar environment.

Precision is measured as the scatter of the estimated-to-true ratio 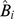 which is visualized by the B & W plots (Fig. 1). A box ranging from 0.8 to 1.2 means that 50% of the estimated numbers of collisions were within ±20% of the true value. The precision of the “corrected count” method had the highest value possible (white boxes in Fig. 1) because we assumed that the true detection probability was known (the large scatter is only due to the stochasticity of the search process). Comparing the precision obtained by the “corrected count” method with that obtained from our model suggests that the model estimates are at least as precise as the “corrected count”.

As expected, predictions for new turbines (of which the data was not used to fit the model to) are less precise than estimates for the actual data. Also, predictions for new turbines are much more precise when the model has been fitted to a large set of data.

To summarize, mortality estimates from our model seem to be unbiased, the precision of the model estimates is at least as high as that of the “corrected count” estimator, and the models can reliably predict the number of fatalities for new data without carcass searches.

### Application of the Model to Real Data

When we fitted the model to the real data, the posterior predictive distribution of the number of carcasses found did not deviate from the observed distribution of carcasses found (Tables 5, 6). In particular, it does not seem to be necessary to use a model that allows for zero-inflation or overdispersion since the percentage of zeros in the data can be precisely predicted by our models and the range of the observed number of carcasses found corresponds to the range of the data simulated by the model.

**Table 5:** Observed frequencies of the numbers of carcasses found per search in the 2007 data set compared to the ones predicted from our two models (see text).

Number of carcasses found	0	1	2	3	4
observed frequency	453	18	2	0	0
predicted by 1-level observation model	453 (445–458)	18 (11–25)	3 (0–4)	0 (0–0)	0 (0–0)
predicted by 3-level observation model	449 (441–456)	22 (14–29)	2 (0–5)	0 (0–0)	0 (0–0)

The ranges are 95% prediction intervals for the model predictions.

**Table 6:** Observed frequencies of the numbers of carcasses found per search in the 2008 data set compared to the ones predicted from our two models (see text).

Number of carcasses found	0	1	2	3	4
Observed	1191	33	1	0	0
predicted by 1-level observation model	1192 (1182–1200)	32 (23–41)	1 (0–2)	0 (0–0)	0 (0–0)
predicted by 3-level observation model	1193 (1183–1200)	31 (23–39)	1 (0–3)	0 (0–0)	0 (0–0)

The ranges are 95% prediction intervals for the model predictions.

**Figure 2:**
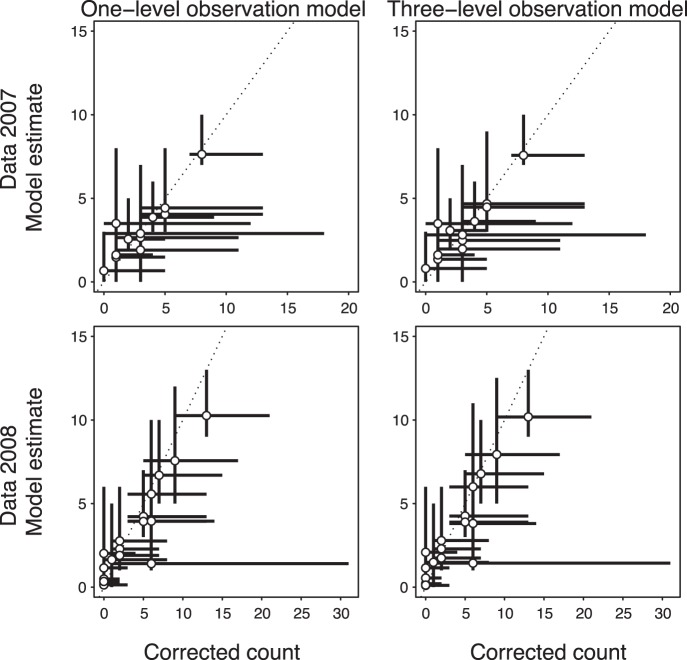
Estimated number of collisions for each turbine in the two data sets (2007 and 2008) based on the one- and three-level observation model vs. the conventional “corrected count” estimate. Segments give the 95% credible intervals. The dotted line indicates perfect coincidence.

In our field data set, we found a positive correlation of acoustic activity and the number of bat collisions in both data sets. Wind speed had a negative quadratic effect in both data sets and all models. The maximum collision rate (given constant acoustic activity) was estimated for wind speeds of 4.3 and 5.7 m/s in the 2007 data sets for the one- and three-level model respectively, and for 3.5 m/s in the 2008 data for both models (Table 4). We estimated an average of 0.05 to 0.08 bats killed per turbine and night. These estimates correlate with the estimate obtained by the conventional “corrected count” method (Table 4, Fig. 2).

## Discussion

### Bias and Precision of Mortality Estimation and Predictive Power of the Model

We have shown how information about carcass detection probability can be integrated in a model that relates animal density measurements to the number of carcasses found to get an unbiased mortality estimate and to predict the collision rate. The precision of the model estimates is at least as high as when simply “correcting” the number of carcasses as is usually done [16]–[18], [35], [36].

Both methods (our two models and the conventional “corrected count method”) rely on the information available for detection probabilities, i.e. if the estimate of detection probability is biased then this bias will propagate into both estimators in a similar way. The methods required to obtain an unbiased and precise estimate for carcass detection probability *p* have been addressed by many authors, e.g. [17], [18], [22], [37], [38] and is not the aim of our study. Here, we assume that an unbiased estimate 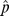 is available. The purpose of our study was to present a method to combine detection probability 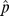 and the number of carcasses found with further predictors for the collision rate such as wind speed and acoustic activity data to obtain a more precise mortality estimate. More importantly, by doing so, we obtained a model that relates the predictor variables to the collision rate allowing for a prediction of collisions in a new setting (i.e. for new turbines) and with no need for information from carcass searches for this new setting.

It is important to be aware that the precision of any mortality estimate depends primarily on the total number of collisions in the data set. This makes comparisons between different (simulation) studies difficult. For example, no “corrected count” estimate carried out by [17] for simulated data exceeded a bias of ±27%. In contrast, our estimated bias for the same “corrected count” estimator ranged from minus infinity to ∼+300% (Fig. 1). This was true even though in our study, detection probability *p* was assumed to be known without bias, whereas in [17] *p* was estimated including a potential bias (the aim of [17] was to estimate this bias). Our results show the highest precision possible when mortality is estimated based on carcass searches and an estimated detection probability 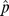. The precision in [17] was higher than in our study, because the data simulated by [17] contained a higher total number of collisions per turbine (∼120) than our simulated data (mean was 4.5 in the data sets with 100 days per turbines and 14.2 in the data sets with 500 days per turbine). The scatter shown in Fig. 1 reflects the stochasticity of the carcass detection process.

Our simulations show that it is possible to use our models to predict collision rates for new turbines where no carcass searches have been carried out, but it also shows that it is important to fit the model to a sufficiently large data set. Such predictions assume that the relationship between the predictor variables and collision rate for the new turbine is similar to the turbines where the data was collected to fit the model. How well this assumption is met depends on the data at hand. Therefore, it is impossible to give a general measure for the predictive power of the model. Generally, the predictive power will depend on how much information about the collision rate is contained in the predictor variables. In our example data, the combination of acoustic bat activity measured at the nacelle of the turbine and wind speed appears to provide strong information about the bat collision rate as has been shown in earlier studies [25], [39]. As we tried to imitate reality as closely as possible in our simulation study, the results indicate that predictions about new turbines seem to be possible with reasonable precision (Fig. 1).

The strength of our model is that it allows for quantitative predictions of the collision rate. Most of the models hitherto used to mitigate bat collisions at wind turbines predict the bat collision rate qualitatively, e.g. as a probability of bats being active [40] or as an index of bat activity [41]. Such qualitative models allow for a mitigation of collisions, but they do not allow the effectiveness of the mitigation strategies to be measured since they provide no information on how the bat activity indices transform quantitatively into collision rates. A collision model has recently been developed that enables quantifying collision rates for Red Kites *Milvus milvus* based on the distance between the wind turbine and the aerie of the birds [42]. This model is based on physical characteristics of the wind turbines and behavioural parameters of the Red Kite such as flight height, flight frequency in relation to the distance to the aerie, and behavioural reactions to obstacles such as wind turbines. To use such a model, animal flight behaviour has to be known in detail. For many bird and bat species flight behaviour is not known in such detail. Our model provides a method to obtain quantitative estimates and predictions of collision rates even if we know little about the exact flight behaviour of the animals.

### Application of the Model to Real Data

We found an increasing number of bat collisions with increasing acoustic bat activity per night. The higher the number of bats which fly in the rotor-swept area of a turbine, the higher the number of bats which can potentially collide with the rotor blades. This relationship is undisputed and has been reported several times [12], [24], [25].

In all models, wind showed a negative quadratic effect with the maximal collision rate between 2 and 6 m/s. Below 2 m/s the rotor blades do not usually move and collision rate is, therefore, close to zero. On the other hand, bat activity was low at wind speeds above 6 m/s which explains the decreasing estimated collision rates at high wind speeds. It is known that bat activity correlates negatively with wind speed [40]. The negative correlation of bat activity and wind speed has been used to mitigate the bat collision rate by increasing the cut-in wind speed of turbines. Fixed cut-in wind speed values, e.g. 5 m/s or 6 m/s, have been defined [43], [44]. Our results corroborate these cut-in wind speeds in that they predicted small collision rates when wind speeds were above 6 m/s.

Our model simultaneously predicts collision rates based on acoustic activity and wind speed. Therefore, the wind speeds with maximum collision rates (3.5–5.7 m/s) are not the univariate wind speeds expected to correlate best with maximum collision rates. Bat activity decreases with increasing wind speed and above 5 m/s bat activity is far below average. Because of the strong correlation of wind speed and acoustic activity (i.e. collinearity) it was not possible to fully separate the effects of wind and activity on collision rate. However, predictions based on realistic pairs of wind speed and activity measurements are not affected by collinearity provided the model fit is good [45], which was the case in our example.

The average number of bat collisions during the three months of July, August and September in our data set was estimated to be 7.4 per turbine in 2007 and 4.3 in 2008. These estimates are in line with other studies in Central and Western Europe. For example, [35] obtained an estimate of 6 to 26 bats killed annually per turbine in France and a review on bat mortality for north-western European countries came up with 0 to 20 bats killed annually per turbine [15]. The difference in the estimated average number of collisions between the two years in our data set may be due to a random sampling error, different turbines being sampled, or the between-year variance in the number of fatalities. Even if the number of nights sampled here was large, the effective sample size, i.e. the number of carcasses found, was low. This emphasizes the need for large and long-term sampling efforts if the aim is to assess between-year variance in fatality rates.

The total mortality estimates from the models did not differ from those obtained by the “corrected count” method. This is not surprising because we used the same detection probabilities for both methods. However, when the number of carcasses found is low or even zero (for example, when estimating the number of fatalities for each turbine separately), the “corrected count” is very sensitive to the stochastic detection process, i.e. if by chance one more carcass is found, the estimate changes substantially. In contrast, our model incorporates the information from acoustic bat activity and wind speed to estimate the collision rate for wind turbines where no or only few carcasses have been found. Therefore, we think that turbine-specific mortality estimates are more reliable if taken from the model rather than obtained by a deterministic formula, given that the model can be fitted to a large enough data set.

### Applications of the Model

The model allows the prediction of collision rates based on variables that correlate with the collision rate. In our example we used data on acoustic activity and wind speed. When estimating bird collision rates, bird density measurements, such as migration traffic rates measured by radar [46], [47] or infrared cameras [48], and other predictors of bird collision rates (e.g. wind velocity and visibility [49]) may be used. However, the model may be used without any predictor variable. In this case, the model will assume a constant collision rate over time. As a consequence, the predicted collision rate for new turbines or new days will be estimated based on the average collision rate in the data at hand. Therefore, without predictors the model can be used for the same purpose as the conventional “corrected count” method [17], [18] except that prediction intervals can be obtained from the model given the variance structure is appropriate. Assessment of the appropriateness of the variance structure can be done by posterior predictive model checking as was the case here. If the real data shows a higher variance or a higher proportion of zeros than the data simulated from the model, an extra variance parameter or a zero-inflation model structure can be included in the model.

The method we presented here has two advantages over the “corrected count” method:

1. The model provides an estimate for the collision rate for every single day whereas the conventional methods only permit an estimate of total numbers of collisions summed over a large time span or over several turbines. Consequently, the model provides the ability to assess the influence of factors varying over time or space on the collision rate. For example, we also applied the model to estimate the influence of rotor diameter on the collision rate of Red Kites (Bellebaum unpublished data), and to investigate the effect of different curtailment strategies (own unpublished data).
2. Since our method is model based, it also allows for a prediction of the collision rate for new turbines and/or nights without carcass searches, if informative predictors are used (such as the acoustic activity and wind speed we used in our case study) and the new turbines or nights are similar to the ones used for model fitting. The model can therefore be used to develop curtailment algorithms.

Predictions of bat collision rates from our specific models presented in Table 4 can be made only for turbines or nights that are similar to those in our data sets. Turbine type, rotor diameter, species composition, activity patterns, wind conditions, bat detector types and sensitivity need to be similar enough to those from our field study. To apply the model in other conditions, new training data would need to be collected. It may be desirable to pool many such data files to develop a model that allows for a prediction of the collision rate for different turbine types at different places in the world and for different species or groups of species. Such collaboration would be particularly valuable because of the large sample sizes required and the effort needed to obtain such data.

Predicting collision rate from variables that are easy to measure, such as wind speed, is helpful e.g. for sites that are difficult to assess by carcass searches due to high scavenger removal rates (that are commonly found in Central Europe [38]) or due to large areas that cannot be searched, as is often the case in forest areas or off-shore. Also, carcass searches are time-consuming and therefore expensive. Our model can help to reduce the cost of estimating the number of collisions and is therefore a useful tool for the long-term monitoring of bat and bird collisions at wind turbines. Also, the prediction of the collision rate from easy to measure predictors can be implemented in curtailment algorithms that reduce the collision rate at a minimum loss of energy production [50].

In summary, we have developed a stochastic model that combines carcass search data with additional predictors for the collision rate to estimate the number of collisions of animals at wind turbines. This model can be used to evaluate the collision rates at wind energy turbines, to investigate factors affecting the collision rate, and to predict the number of collisions for new turbines or new days without carcass searches.

## Supporting Information

File S1 **WinBUGS code for the one-level and three-level observation model.**
(DOCX)

File S2 **R-Code for the data simulation.**
(DOCX)
