# Supplementary material for: Estimating Bat and Bird Mortality Occurring at Wind Energy Turbines from Covariates and Carcass Searches Using Mixture Models
Source: PLoS One. 2013 Jul 3;8(7):e67997. doi: 10.1371/journal.pone.0067997 (PMC3700871; doi:10.1371/journal.pone.0067997)
Supplement: File S1 — WinBUGS code for the one-level and three-level observation model. (DOCX) [file pone.0067997.s001.docx]

**Supplementary file S1: WinBUGS-Code for the one-level and three-level observation models**

#--------------------------------------------------------------------------------------------------------------------------

# one-level observation model

#---------------------------------------------------------------------------------------------------------------------------

# data

nsites number of sites (turbines), number

Ti number of days (nights) sampled per site, vector of length nsites

act acoustic activity measures (log-transformed and standardized),

matrix of dimension nsites x max(Ti)

wind wind speed data (standardsized), matrix of dimension nsites x max(Ti)

y number of carcasses found, matrix of dimension nsites x max(Ti)

a Parameter alpha of Beta distribution describing the information on the carcass detection

probability, vector of length nsites

b Parameter alpha of Beta distribution describing the information on the carcass detection

probability, vector of length nsites

# parameters

N number of freshly killed animals per day (night) and site (turbine),

matrix of dimension nsites x max(Ti)

lambda expected number of freshly killed animals per day (night) and site (turbine),

matrix of dimension nsites x max(Ti)

alpha0 model coefficient, number

alpha1 model coefficient, number

alpha2 model coefficient, number

alpha3 model coefficient, number

# derived parameters

sumN total number of collisions per site (turbine), vector of length nsites

#---------------------------------------------------------------------------------------------------------------------------

# WinBUGS code

model{

# likelihood

for (i in 1:nsites){

for(t in 1:Ti[i]){

N[i,t] ~ dpois(lambda[i,t])

lambda[i,t]<-exp(alpha0+alpha1*act[i,t]+ alpha2*wind[i,t] + alpha3*pow(wind[i,t],2))

y[i,t]~dbin(p[i], N[i,t])

}

}

# priors

for(i in 1:nsites){

p[i]~dbeta(a[i],b[i]) # informative prior for detection probability
}

alpha0~dnorm(0, 0.01)

alpha1~dnorm(0, 0.01)

alpha2~dnorm(0, 0.01)

alpha3~dnorm(0, 0.01)

# derived parameters

for(i in 1:nsites){

sumN[i]<-sum(N[i, 1:Ti[i]]) # total number of collisions per turbine

}

}

#---------------------------------------------------------------------------------------------------------------------------

# three-level observation model

#---------------------------------------------------------------------------------------------------------------------------

# data

nsites number of sites (turbines), number

Ti number of days (nights) sampled per site, vector of length nsites

act acoustic activity measures (log-transformed and standardized),

matrix of dimension nsites x max(Ti)

wind wind speed data (standardsized), matrix of dimension nsites x max(Ti)

y number of carcasses found, matrix of dimension nsites x max(Ti)

f.a Parameter alpha of Beta distribution describing the information on the searcher efficiency,

vector of length nsites

f.b Parameter alpha of Beta distribution describing the information on the searcher efficiency

vector of length nsites

s.a Parameter alpha of Beta distribution describing the information on the persistence

probability, vector of length nsites

s.b Parameter alpha of Beta distribution describing the information on the persistence

probability, vector of length nsites

a proportion of carcasses lying in the searched area, vector of length nsites

# parameters

N number of freshly killed animals per day (night) and site (turbine),

matrix of dimension nsites x max(Ti)

lambda expected number of freshly killed animals per day (night) and site (turbine),

matrix of dimension nsites x max(Ti)

alpha0 model coefficient, number

alpha1 model coefficient, number

alpha2 model coefficient, number

alpha3 model coefficient, number

Nfar number of carcasses falling in the searched area, matrix of dimension nsites x max(Ti)

Narea number of carcasses being in the searched area (temporary auxiliary variable),

matrix of dimension nsites x max(Ti)

Nrem number of carcasses that have been killed during earlier searches and that remain on the

searched area because they have not been removed by scavengers or searchers,

matrix of dimension nsites x max(Ti)

# derived parameters

sumN total number of collisions per site (turbine), vector of length nsites

#---------------------------------------------------------------------------------------------------------------------------

# WinBUGS code

model{

# likelihood

for (i in 1:nsites){

N[i,1] ~ dpois(lambda[i,1]) # first night

Nfar[i,1]~dbin(a[i], N[i,1]) # number of carcasses falling in searched area

Narea[i,1] <- Nfar[i,1] # number of carcasses being in searched area

Nrem[i,1]~dbin(s[i], Narea[i,1]) # number of carcasses remaining in searched area

y[i,1]~dbin(f[i], Nrem[i,1])

for(t in 2:Ti[i]){

N[i,t] ~ dpois(lambda[i,t])

Nfar[i,t]~dbin(a[i], N[i,t]) # number of carcasses falling in searched area

Narea[i,t] <- Nfar[i,t] + Nrem[i,t-1]-y[i,t-1] # number of carcasses being in searched area

Nrem[i,t]~dbin(s[i], Narea[i,t]) # number of carcasses remaining in searched area

y[i,t]~dbin(f[i], Nrem[i,t])

}

}

for(i in 1:nsites){

for(t in 1:Ti[i]){

lambda[i,t]<-exp(alpha0+alpha1*act[i,t]+ alpha2*wind[i,t] + alpha3*pow(wind[i,t],2))

}

}

# priors

for(i in 1:nsites){

f[i]~dbeta(f.a[i],f.b[i]) # informative prior for searcher efficiency

s[i]~dbeta(s.a[i], s.b[i]) # informative prior for persistence probability

}

alpha0~dnorm(0, 0.01)

alpha1~dnorm(0, 0.01)

alpha2~dnorm(0, 0.01)

alpha3~dnorm(0, 0.01)

# derived parameters

for(i in 1:nsites){

sumN[i]<-sum(N[i,1:Ti[i]]) # total number of collisions per turbine

}

}
