# Supplementary material for: Estimating Bat and Bird Mortality Occurring at Wind Energy Turbines from Covariates and Carcass Searches Using Mixture Models
Source: PLoS One. 2013 Jul 3;8(7):e67997. doi: 10.1371/journal.pone.0067997 (PMC3700871; doi:10.1371/journal.pone.0067997)
Supplement: File S2 — R-Code for the data simulation. (DOCX) [file pone.0067997.s002.docx]

**Supplementary file S2**: R-Code to simulate carcass search data and fit the mixture models

#-------------------------------------------------------------------------

# load data

load("ready.RData")

# contains:

# “detdat2”: Table 2

# “Akt”: vector with acoustic bat activity

# “Wind”: vector with wind measurements

# “Anl”: vector with index of turbine (turbine ID)

# “alpha0post”: random draws from the posterior distribution of alpha0

# “alpha1post”: random draws from the posterior distribution of alpha1

# “alpha2post”: random draws from the posterior distribution of alpha2

# “alpha3post”: random draws from the posterior distribution of alpha3

# “nsimalpha”: number of random draws from the posterior distr. of alpha

# load packages

library(carcass)

library(R2WinBUGS)

#---------------------------------------------------------------------------

# transform 95% CI into shapeparameter of beta-distribution

detdat2$f.a <- shapeparameter(detdat2$f, detdat2$f.lower, detdat2$f.upper)$a

detdat2$f.b <- shapeparameter(detdat2$f, detdat2$f.lower, detdat2$f.upper)$b

#---------------------------------------------------------------------------

bugsworkingdir <- ".../WinBUGS14" # set directory of WinBUGS

#---------------------------------------------------------------------------

# define models

# one-level observation model

sink("model.txt")

cat("

model{

for (i in 1:nsites){

for(t in 1:Ti[i]){

N[i,t] ~ dpois(lambda[i,t])

lambda[i,t]<-exp(alpha0+alpha1*act[i,t]+ alpha2*wind[i,t] +alpha3*pow(wind[i,t],2))

y[i,t]~dbin(p[i], N[i,t])

}

}

# priors

for(i in 1:nsites){

p[i]~dbeta(a[i],b[i])

}

alpha0~dnorm(0, 0.01)

alpha1~dnorm(0, 0.01)

alpha2~dnorm(0, 0.01)

alpha3~dnorm(0, 0.01)

for(i in 1:nsites){

sumN[i]<-sum(N[i, 1:Ti[i]])

sumlambda[i] <- sum(lambda[i, 1:Ti[i]])

}

for(i in 1:nnewobs){

newlambda[i] <- exp(alpha0+alpha1*newact[i]+alpha2*newwind[i] +

alpha3*pow(newwind[i], 2))

newN[i]~dpois(newlambda[i])

}

sumnewN <- sum(newN[1:nnewobs])

}

",fill=TRUE)

sink()

# three-level observation model

sink("modelobs.txt")

cat("

model{

for (i in 1:nsites){

N[i,1] ~ dpois(lambda[i,1])

Nfar[i,1]~dbin(a[i], N[i,1])

Narea[i,1] <- Nfar[i,1]

Nrem[i,1]~dbin(s[i], Narea[i,1])

y[i,1]~dbin(f[i], Nrem[i,1])

for(t in 2:Ti[i]){

N[i,t] ~ dpois(lambda[i,t])

Nfar[i,t]~dbin(a[i], N[i,t])

Narea[i,t] <- Nfar[i,t] + Nrem[i,t-1]-y[i,t-1]

Nrem[i,t]~dbin(s[i], Narea[i,t])

y[i,t]~dbin(f[i], Nrem[i,t])

}

}

for(i in 1:nsites){

for(t in 1:Ti[i]){

lambda[i,t]<-exp(alpha0+alpha1*act[i,t]+ alpha2*wind[i,t] +alpha3*pow(wind[i,t],2))

}

}

# priors

for(i in 1:nsites){

f[i]~dbeta(f.a[i],f.b[i])

s[i]~dbeta(s.a[i], s.b[i])

}

alpha0~dnorm(0, 0.01)

alpha1~dnorm(0, 0.01)

alpha2~dnorm(0, 0.01)

alpha3~dnorm(0, 0.01)

for(i in 1:nsites){

sumN[i]<-sum(N[i,1:Ti[i]])

sumlambda[i] <- sum(lambda[i,1:Ti[i]])

}

for(i in 1:nnewobs){

newlambda[i] <- exp(alpha0+alpha1*newact[i]+alpha2*newwind[i] +

alpha3*pow(newwind[i], 2))

newN[i]~dpois(newlambda[i])

}

sumnewN <- sum(newN[1:nnewobs])

}

",fill=TRUE)

sink()

#----------------------------------------------------------------------------------

# prepare data.frame to store the results

settings <-c(100, 300)

nset <- length(settings)

R<-50

scAkt <- as.numeric(scale(log(Akt+1)))

scWind <- as.numeric(scale(Wind))

trueN <- array(dim=c(nsites, R, nset))

estN <- array(dim=c(nsites, R, nset))

estNobs <- array(dim=c(nsites, R, nset))

trueNnew <- matrix(ncol=R, nrow=nset)

estNnew <- matrix(ncol=R, nrow=nset)

estNnewobs <- matrix(ncol=R, nrow=nset)

count <- array(dim=c(nsites, R, nset))

rhat <- array(dim=c(nsites, R, nset))

rhatobs <- array(dim=c(nsites, R, nset))

n.effobs <- array(dim=c(nsites, R, nset))

n.eff <- array(dim=c(nsites, R, nset))

for(setting in 1:nset){

npsite <- settings[setting]

for (r in 1:R){

y <- matrix(nrow=nsites, ncol=npsite)

N <- matrix(nrow=nsites, ncol=npsite)

Nfar <- matrix(nrow=nsites, ncol=npsite)

Narea <- matrix(nrow=nsites, ncol=npsite)

Nrem <- matrix(nrow=nsites, ncol=npsite)

windmat <- matrix(nrow=nsites, ncol=npsite)

actmat <- matrix(nrow=nsites, ncol=npsite)

Ti <- rep(npsite, nsites)

for(i in 1:nsites){

index <- sample(1:sum(Anl==i), npsite, replace=TRUE)

windmat[i, 1:Ti[i]] <- scWind[Anl==i][index]

actmat[i, 1:Ti[i]] <- scAkt[Anl==i][index]

}

randalpha <- sample(1:nsimalpha, 1)

alpha0<-alpha0post[randalpha]

alpha1<-alpha1post[randalpha]

alpha2<-alpha2post[randalpha]

alpha3<-alpha3post[randalpha]

# simulate data

lambda<-exp(alpha0 + alpha1*actmat + alpha2*windmat + alpha3*windmat^2)

for(i in 1:nsites){

N[i,] <- rpois(npsite, lambda[i,]) # killed at i in t

Nfar[i,] <- rbinom(npsite, size=N[i,], prob=min(detdat2$fkf[i],1))

Narea[i,1] <- Nfar[i,1]

Nrem[i,1] <- rbinom(1, size=Narea[i,1], prob=detdat2$S[i])

y[i,1] <- rbinom(1, size=Nrem[i,1], prob=detdat2$f[i])

for(t in 2:npsite) {

Narea[i,t] <- Nfar[i,t] + Nrem[i,t-1] - y[i,t-1]

Nrem[i,t] <- rbinom(1, size=Narea[i,t], prob=detdat2$S[i])

y[i,t] <- rbinom(1, size=Nrem[i,t], prob=detdat2$f[i])

}

}

# site to be left out (for cross-validation)

k <- sample(1:nsites, 1)

newact <- actmat[k,] # activity measures for new site

newwind <- windmat[k,] # wind measures for new site

trueNnew[setting, r] <- sum(N[k,])

act <- actmat[-k,]

wind <- windmat[-k,]

# save true values

trueN[-k,r, setting] <- apply(N[-k,], 1, sum)

count[-k,r,setting] <- apply(y[-k,], 1, sum)

# bundle data for WinBUGS

datax<-list(y=y[-k,], Ti=Ti[-k], act=act, wind=wind, nsites=nsites-1, a=detdat$a[-k], b=detdat$b[-k],newact=newact, newwind=newwind, nnewobs=npsite)

dataxobs <- list(y=y[-k,], Ti=Ti[-k], act=act, wind=wind, nsites=nsites-1, a=detdat2$fkf[-k], f.a=detdat2$f.a[-k],f.b=detdat2$f.b[-k],s.a=detdat2$S.a[-k],s.b=detdat2$S.b[-k], newact=newact, newwind=newwind, nnewobs=npsite)

inits <- function(){

list(p=rbeta(nsites-1, datax$a, datax$b), alpha0 = rnorm(1, -3, 0.4), alpha1=rnorm(1, 0.1, 0.3), alpha2=rnorm(1, 0.1, 0.3), alpha3=rnorm(1, 0.1, 0.3), N=datax$y+1, newN=rpois(npsite, 1)+1)

}

initsobs <- function(){

list(f=rbeta(nsites-1, dataxobs$f.a, dataxobs$f.b), alpha0 = rnorm(1, -3, 0.4), alpha1=rnorm(1, 0.1, 0.3), alpha2=rnorm(1, 0.1, 0.3), alpha3=rnorm(1, 0.1, 0.3),

N=dataxobs$y +3, Nfar=dataxobs$y +2,Nrem=dataxobs$y+1, s=rbeta(nsites-1, dataxobs$s.a, dataxobs$s.b))

}

# MCMC settings

nc <- 2 ; nb <- 1000 ; ni <- 19000 ; nt <- 9

params <- c("sumN", "sumlambda", "sumnewN")

# Start Gibbs sampler

mod <- bugs(datax, inits, params, "model.txt", n.chains=nc, n.iter=ni, n.burn = nb, n.thin=nt, debug = FALSE, bugs.directory=bugsworkingdir)

estN[-k,r, setting] <- mod$summary[1:(nsites-1), "mean"]

estNnew[setting, r] <- mod$summary["sumnewN", "mean"]

rhat[-k, r, setting] <- mod$summary[1:(nsites-1), "Rhat"]

n.eff[-k, r, setting] <- mod$summary[1:(nsites-1), "n.eff"]

save(trueN, file="trueN.RData")

save(estN, file="estN.RData")

save(trueNnew, file="trueNnew.RData")

save(estNnew, file="estNnew.RData")

save(count, file="count.RData")

save(rhat, file="rhat.RData")

save(n.eff, file="n.eff.RData")

mod <- bugs(dataxobs, initsobs, params, "modelobs.txt", n.chains=nc, n.iter=25000, n.burn = 6000, n.thin=nt, debug = FALSE, bugs.directory=bugsworkingdir)

estNobs[-k,r, setting] <- mod$summary[1:(nsites-1), "mean"]

rhatobs[-k, r, setting] <- mod$summary[1:(nsites-1), "Rhat"]

n.effobs[-k, r, setting] <- mod$summary[1:(nsites-1), "n.eff"]

estNnewobs[setting, r] <- mod$summary["sumnewN", "mean"]

save(estNobs, file="estNobs.RData")

save(estNnewobs, file="estNnewobs.RData")

save(rhatobs, file="rhatobs.RData")

save(n.effobs, file="n.effobs.RData")

cat("Setting", setting, "simulation", r, "of", R, "\n\n")

} # close simulation r

} #close setting

################################################################################
